# A Whole Genome Re-Sequencing Based GWA Analysis Reveals Candidate Genes Associated with Ivermectin Resistance in *Haemonchus contortus*

**DOI:** 10.3390/genes11040367

**Published:** 2020-03-28

**Authors:** Sawar Khan, Ayesha Nisar, Jianqi Yuan, Xiaoping Luo, Xueqin Dou, Fei Liu, Xiaochao Zhao, Junyan Li, Habib Ahmad, Sardar Azhar Mehmood, Xingang Feng

**Affiliations:** 1 Shanghai Veterinary Research Institute, Chinese Academy of Agricultural Sciences, Key Laboratory of Animal Parasitology, Ministry of Agriculture of China, Shanghai 200241, China; 2 Veterinary Research Institute, Inner Mongolia Academy of Agricultural and Animal Husbandry Sciences, Hohhot 010031, China; 3 Department of Genetics, Hazara University, Mansehra 21300, Pakistan; 4 Department of Zoology, Hazara University, Mansehra 21300, Pakistan

**Keywords:** *Haemonchus contortus*, population genomics, drug resistance, ivermectin, RNAi, functional validation, larvae culture

## Abstract

The most important and broad-spectrum drug used to control the parasitic worms to date is ivermectin (IVM). Resistance against IVM has emerged in parasites, and preserving its efficacy is now becoming a serious issue. The parasitic nematode *Haemonchus contortus* (Rudolphi, 1803) is economically an important parasite of small ruminants across the globe, which has a successful track record in IVM resistance. There are growing evidences regarding the multigenic nature of IVM resistance, and although some genes have been proposed as candidates of IVM resistance using lower magnification of genome, the genetic basis of IVM resistance still remains poorly resolved. Using the full magnification of genome, we herein applied a population genomics approach to characterize genome-wide signatures of selection among pooled worms from two susceptible and six ivermectin-resistant isolates of *H. contortus*, and revealed candidate genes under selection in relation to IVM resistance. These candidates also included a previously known IVM-resistance-associated candidate gene HCON_00148840, glc-3. Finally, an RNA-interference-based functional validation assay revealed the HCON_00143950 as IVM-tolerance-associated gene in *H. contortus*. The possible role of this gene in IVM resistance could be detoxification of xenobiotic in phase I of xenobiotic metabolism. The results of this study further enhance our understanding on the IVM resistance and continue to provide further evidence in favor of multigenic nature of IVM resistance.

## 1. Introduction

Helminthic parasites constitute an important parasitic group that has major impacts on public health, veterinary, agriculture, and socioeconomic sectors worldwide. They are able to infect humans, animals, and plants. With extreme diversity, helminths are able to cause significant morbidity and mortality in their hosts. They infect about three billion people across the globe, and are responsible for highest economic losses in livestock due to lower productivity [1]. Control of these parasites of public health and veterinary importance is almost entirely dependent on the large-scale administration of anthelmintic drugs such as macrocyclic lactones, salicylanilides, and benzimidazoles. The gastrointestinal parasitic strongylid nematode *Haemonchus contortus* (Rudolphi, 1803) is an important worm, which primarily infects the goats, sheep, and cattle [2]. It is a blood-feeding parasite that leads to a pathological condition called haemonchosis, which is characterized by a series of symptoms, including anemia, diarrhea, weight loss, or even death in case of severe infection. Damages due to haemonchosis can lead to billions of economic losses to the breeding industry of small ruminants [3,4,5].

Ivermectin (IVM) is a broad-spectrum antiparasitic drug that has been extremely successful in the control of a number of helminths of public health and veterinary importance [6]. However, the extensive and uncontrolled use of IVM in livestock treatment has led to widespread resistance in gastrointestinal nematodes, such as *H. contortus* [7,8]. Alongside of veterinary sector where the rapid emergence of resistance against both single and multiple drug classes has been widely reported [7,9], there are also growing concerns regarding the emergence of resistance against the drugs used in helminths control in human [10,11,12]. The problem of IVM resistance has also been reported for pests [10,13,14,15]. Such resistance has a greater impact on public health as well as on livestock production system [16], and is a growing issue in all across the globe that requires immediate attention [17,18]. Therefore, preserving the efficacy of IVM is becoming a serious issue now. *H. contortus* has a successful track record in anthelmintic resistance, and it has shown multidrug resistance including IVM [8,19]. The improper management and use of sublethal doses of drugs led to the rapid selection and adaptation of parasites against the drug. So, there is a dire need of discovering useful genetic markers and variants associated with drug resistance in parasitic nematodes [20]. Discovery of such genetic markers would help to counterbalance the impact of anthelmintic resistance and will aid in development of the next generation of anthelmintic drugs [21].

The IVM resistance in *H. contortus* is likely to be multigenic in nature [22,23]. However, conventional methods or strategies could hardly characterize a few of these genes [21]. So far, the research on IVM resistance has been dominated by a number of individual candidate genes studies [21]. Some genes were proposed as candidate of IVM resistance based on comparison of haplotypes or SNPs’ allelic frequencies between the small numbers of susceptible and resistant isolates [24,25,26,27,28] or before and after the IVM treatment [29,30]. However, none of these proposed genes was functionally validated so far. Efforts for validation of a number of these candidate genes have shown no association of the tested genes to IVM resistance [31,32]. This lack of consistency and discordance among the studies accompanied by extremely high level of genetic diversity of *H. contortus* has led to much discussion on the complexity of the genetic basis of IVM resistance [21,33,34,35]. At the advent of high-throughput sequencing technology and enrichment of genomic resources of *H. contortus* [36,37,38,39], the genome-wide approaches (for characterizing drug resistance) are now feasible and are emerging for *H. contortus* [34,40,41,42,43,44]. Under population genomics approach, genome-wide scans for potential SNPs and their allele frequencies across the IVM-susceptible and resistant populations of *H. contortus* can reveal the IVM-resistance-associated variants. The whole genome re-sequencing (WGS) provides full magnification of the genome, and thus offers a potential mean for: analyzing each and every part of genome, bulk analysis of the pools of resistant and sensitive worm populations, and analyzing variations in all the genes at each and every nucleotide position across the pooled data. In this study, we aimed to explore the IVM-resistance-associated candidate genes in *H. contortus* by applying population genomics approach at the full magnification of genome. 

To this end, we herein applied the WGS to IVM-resistant and IVM-susceptible worms, and analyzed their genome at the full magnification. We first pooled the samples of worms, sequenced the whole genome, and performed variant calling across all the strains. Next, we applied various populations genomics tools to perform genome-wide pairwise analyses of all the strains. Subsequently, we characterized genetic diversity, mapped the analyzed data at individual chromosome level, captured the trends of data, and identified the outliers’ regions across all the chromosomes. Further scanning of the outliers’ regions, and their functional annotations, revealed that candidate genes putatively associated with IVM resistance in *H. contortus*. Finally, the functional validation assay revealed the HCON_00143950 as IVM tolerance-associated gene in *H. contortus*.

## 2. Materials and Methods

### 2.1. Background of Strains, Worm Collection, and Identification 

Adult worms (both male and female) from populations of eight different strains of *H. contortus* (collected from sheep) were used in this study (Table 1). Worms of six of the strains were collected from abomasa of sheep, at different farms in China [5]. Phenotypically, one of the Chinese strains (SXS) was sensitive to IVM and five (WMR, WSR1, WSR2.1, WSR2.2, and SXR) were IVM-resistant. Besides these local Chinese strains, worm samples from two other global strains, one IVM-sensitive (from Australia, ASS) and one IVM-resistant (from UK, UKR), were also included in the study. After obtaining from UK (Moredun Institute) and Australia, both of the strains were maintained in labs in China by serial passage in helminth-free sheep for several years. To differentiate the *H. contortus* samples from closely related species (*H. placei*), the ITS-2 rDNA was sequenced [45]. Screening of SNPs at three conserved sites (24, 205, and 219) established the species of *H. contortus* as described previously [5]. 

### 2.2. DNA Isolation, Library Construction, and Whole Genome Re-Sequencing

For whole genome re-sequencing, pools of male and female adult worms (*n* = 50) were made for each strain of *H. contortus*. Total genomic DNA from each pool sample was isolated through Qiagen DNeasy Blood & Tissue Kit, Hilden, Germany (cat no. 69506), according to its protocol. DNA samples were fragmented using Covaris’ Focused-ultrasonicator S220. Libraries were constructed using TruSeq Nano DNA LT Sample Preparation Kit, San Diego, CA, USA (Illumina, Cat.No.FC-121-4001). Briefly, the DNA fragments were end-repaired, adenylated at 3’ end, and subjected to adapter ligations. The fragments were than enriched, normalized, and finally subjected to paired-end sequencing at Illumina HiSeqXTen platform.

### 2.3. Mapping Reads and Variant Calling

The paired-end Illumina raw reads were first subjected to quality assessment. The software package fastp v0.12.5 [46] was utilized for quality filtering and adapter trimming. The specific criteria for quality filtering were: removing the adaptors sequences, removing the reads with N bases of 5 or more, removing the reads with average base quality score (*Q*) of lower than 20, and removing the reads having length of less than 100 bp. The quality filtered reads were mapped onto the reference genome of *H. contortus* (available online at: ftp://ftp.ebi.ac.uk/pub/databases/wormbase/parasite/releases/WBPS11/species/haemonchus_contortus/PRJEB506/haemonchus_contortus.PRJEB506.WBPS11.genomic.fa.gz) using BWA-0.7.17 [47] with default parameters. Manipulations in the alignment file formats were achieved by SAMTools 1.3.1 [48]. Duplicated reads were marked and removed using Picard (http://broadinstitute.github.io/picard). SNP and InDel callings were performed using haplotype caller module of the GATK4-4.0.3.0 [49]. A base quality of 30 and a minimum of 20 reads depth across a variant was considered. The effects of SNP and InDel were annotated using SnpEff-4.3T [50]. The annotated genome of *H. contortus* [51] was obtained from WormBase ParaSite [52]. Schematic of bioinformatics analysis of sequenced data is presented in Figure F1.2 (Supplementary file F1). 

### 2.4. Characterization of Genetic Variability and Tracing Selection

Within-strain genetic diversity was assessed with nucleotide diversity estimators, Pi (π) and Watterson Theta (θ), in 100 kb windows along the genome using NPStat [53] (code: https://github.com/lucaferretti/npstat). Pileup files as input for NPStat were generated by SAMTools from aligned bam files. Among-strain genetic diversity was assessed by calculating genetic differentiation (Fst) in 5 kb windows along the genome using PoPoolation2 [54]. With SAMTools, mpileup file was generated, which was then converted into a synchronized file in PoPoolation2. This synchronized file was then used as input to calculate pairwise Fst along the genome in 5 kb windows. Significance of pairwise allele comparison was determined using Fisher’s exact test. Neutrality estimator Tajima’s D was calculated in 100 kb windows along the genome using NPStat. The major differentiated windows were further scanned out against the *H. contortus* reference genome assembly to reveal the candidate genes. The retrieved coordinates and genes were searched out at WormBase ParaSite [52] for Gene Ontology (GO) terms and *Caenorhabditis elegans* (Maupas, 1900) orthologues.

### 2.5. Egg Extraction and Culturing Eggs into Larvae

Eggs of *H. contortus* IVM-R (SXR) were extracted from fresh fecal material using a standard salt floatation procedure [55]. Briefly, the fecal samples were homogenized in tap water, and serially poured through 150 > 76 > 68 µm sieve. The filtrate was collected into a 50 mL falcon tube, and was centrifuged at 300× *g* for 2 min. Supernatant was gently discarded, and saturated sodium chloride (NaCl) solution was added to the sediments (tube was filled up to the top). Sample was again centrifuged at 130× *g* for 2 min, and the top layer on the solution containing eggs was transferred with a Pasteur pipette into a new tube. The tube was then filled with distilled water and centrifuged for 2 min at 300× *g*. Supernatant was discarded, and the egg pellet was re-suspended into tap water (1000 eggs/mL). Approximately, 3000–4000 eggs/mL were cultured in a 24 well cell culture plate at 25 °C in a nutritive medium consisting on 1 mL M9 buffer (6 g Na_2_HPO_4_ + 3 g KH_2_PO_4_ + 5 g NaCl + 0.25 g of MgSO_4_7H_2_O per liter of Water) and 100 μL 1 × EBSS until the eggs hatched into first-stage larvae (L1).

### 2.6. RNAi-Based Silencing of Candidate Genes

We selected two (HCON_00143950, Cyt-P450 and HCON_00155510, GPCR) of the candidate genes to be subjected for functional validation using RNAi-based assays in *H. contortus*. This selection was based on the functional annotations of candidates and the feasibility of the validation assay (both for the candidate genes and the available resources at our lab). The Cyt-P450 is known to involve in xenobiotic metabolic processes, whereas the GPCR involve in defense responses and positive regulation of gene expression. Double-stranded siRNA specifically targeting candidate genes (HCON_00143950 and HCON_00155510) were designed using GenScript siRNA Target Finder tool (https://www.genscript.com/tools/sirna-target-finder) by looking/searching for pattern “AAN19” in the ORF of the target gene. All selected siRNA sequences were crossed checked for off-targets by BLAST against the *H. contortus* cDNA database (https://parasite.wormbase.org/Haemonchus_contortus_prjeb506/Tools/Blast). To check the ingestion of siRNA, a nontarget siRNA was also designed from a green fluorescence protein (gfp) sequence, and it was confirmed (nonspecific) by BLAST against the *H. contortus* cDNA database. This nonspecific siRNA was labelled with the fluorochrome-Cy3. All the siRNA sequences (Table S1) were having 3’dTdT overhang and were commercially synthesized from Gene Pharma, Shanghai.

The L2 larvae obtained from in vitro culture of eggs were subjected to gene silencing assays. The RNAi experiments were performed on 3000–4000 L2 larvae in a final volume of 500 μL in 2 mL tubes as described previously [1,56]. First, to investigate the potential ingestion of siRNA constructs for gene silencing experiments, *H. contortus* L2 larvae were incubated (for 2 h at 20 °C under gentle agitation) in a culture medium containing 1 μM of a nonspecific siRNA targeting gfp (nonspecific). Larvae were then washed with water, and siRNA ingestion was checked by fluorescence microscopy. Subsequently, fresh *H. contortus* L2 larvae were incubated (for 48 h at 20 °C under gentle agitation) with siRNA (20 μL of a 20 μM siRNA solution) targeting candidate genes in a final volume of 500 μL in 2 mL tubes.

### 2.7. Larval Feeding Inhibition Assay (LFIA)

The L2 larvae from RNAi-based silencing of candidate genes were incubated (at 25 °C for 2 h) in serial dilutions of IVM, as described previously [57]. IVM was dissolved in DMSO and then diluted with water to desired final concentrations. The IVM-negative control assays were performed with only 1% DMSO. After 2 h of IVM application, the larvae cultures were fed with 10 μL of fluorescein isothiocyanate (FITC)-labelled *Escherichia coli* [58], and tubes were kept incubating overnight at 20 °C. Larvae were then sedimented, and feeding was observed by fluorescence microscopy. Larvae with visible florescence (FITC-labelled *E. coli*) throughout the esophagus and GI tract were counted as fed, while those lacking the florescence in the GI tract, or having it only around the buccal opening, were considered as unfed. All readings were taken in triplicate.

### 2.8. RNA Extraction and qPCR

Total RNA was isolated from the larvae (50 μL pellet) using QIAGEN RNeasy Mini Kit, Hilden, Germany (cat#74104) as per the kit’s protocol. Concentration and purity of isolated RNA were checked by a NanoDrop spectrophotometer (Thermo Scientific, USA). First-strand cDNA synthesis was executed on 1 μg of total RNA using the Takara PrimeScript RT Reagent Kit with gDNA eraser, Kyoto, Japan (cat#RR047A) as per the instructions of kit. The primers for qPCR assays (Table S2) were designed that were targeting the regions external to both the 5’ and 3’ ends of the siRNA-targeted region of the gene, so that the amplification of dsRNA added to the larvae culture can be prevented. The qPCR assays were performed with SYBR Green reagents (YEASEN Hieff^®^, Shanghai, China, cat#11202ES03) by following the kit’s protocol on an Applied Biosystems 7500 instrument in triplicate setup. The relative quantification of the target transcript was analyzed by 2−ΔΔCt method [59].

### 2.9. Statistical Analysis

Percentage of fed larvae was plotted against the logarithm of IVM doses, and the half maximal larval feeding inhibition (LFI50) was calculated. Log (inhibitor) vs. response–variable slope model was applied under nonlinear regression in GraphPad Prism 6 software package. The LFI50 values were compared using extra sum-of-squares *F* test. *P* values of < 0.05 were considered statistically significant. 

### 2.10. Data Availability

Raw sequence data were deposited to CNSA (China National GeneBank Sequence Archive) under the project accession CNP0000811.

## 3. Results

### 3.1. Results of Whole Genome Re-Sequencing

**Figure 1:**
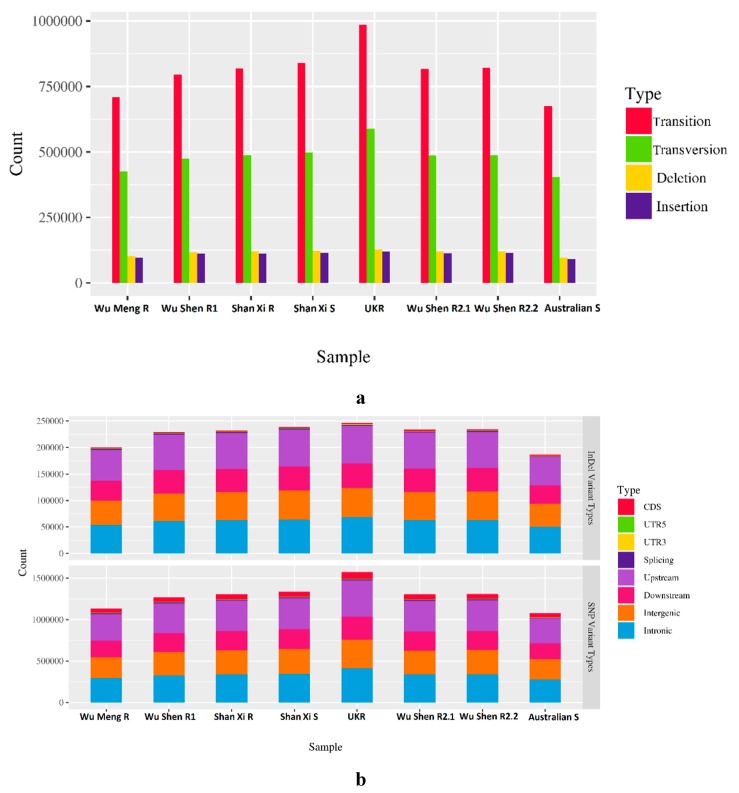
Summary of SNPs and InDel. (**a**) Total numbers of SNPs (transition + transversion) and InDel (deletion + insertion) in each population. (**b**) Distribution of SNPs (bottom panel) and InDel (top panel) across the genome.

Statistics of WGS are presented in an additional file F1. The clean reads ranged in numbers from 135145684 to 164113124 (20.09–24.42 GB). GC content ranged from 42.36% to 43.65%, which is almost equal to that of the previous genome sequencing consortia: 41.3% [20] and 42.4% [37]. About 96% of the bases had a Phred quality score *Q* of >30. To get an alignment for all the downstream analyses, the quality filtered reads were mapped onto the reference genome of *H. contortus*. Mapping rate ranged from 73.48% to 91.05%. Sequencing coverage ranged from 43× to 55.8× across the pools. Total number of SNPs (Indel) were: 1135117(200119) in WMR; 1269230 (229384) in WSR; 1306283 (232273) in SXR; 1338090 (238944) in SXS; 1573743 (246766) in UKR; 1303834 (233877) in WSR2.1; 1308649 (234472) in WSR2.2; and 1079609 (187012) in ASS. On average, a genome-wide distribution of 1 SNP in every 180 to 262 bp was observed all across the populations. In annotations summaries (Figure 1), the upstream and intronic variants dominated across all the strains, while the variations in coding regions were the least of all. Numbers of transitions were higher than numbers of transversions, while the trend of deletions and insertions remained almost similar across the strains.

### 3.2. Genome-wide Analysis of Genetic Diversity and Tracing Selection

**Figure 2:**
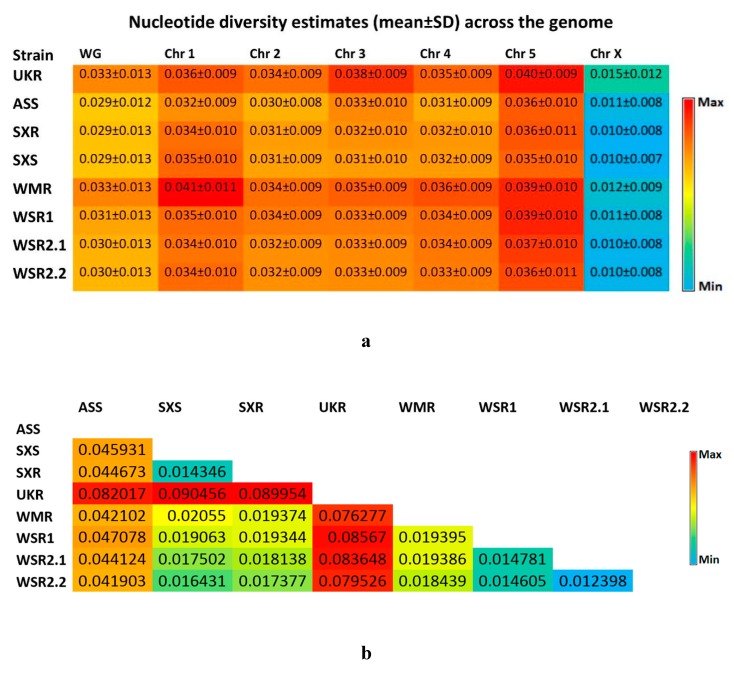
Estimates of genetic diversity. (**a**) Estimates of nucleotide diversity across the genome in all the populations of *Haemonchus contortus* analyzed in the present study. All values are the mean ± SD. (**b**) Genetic differentiation across the populations of *Haemonchus contortus* analyzed in the present study. Genome-wide average Fst scores (fixation index) were calculated in pairwise comparisons. Populations’ codes are according to Table 1. Abbreviation: WG, whole genome; Chr, chromosome.

**Figure 3:**
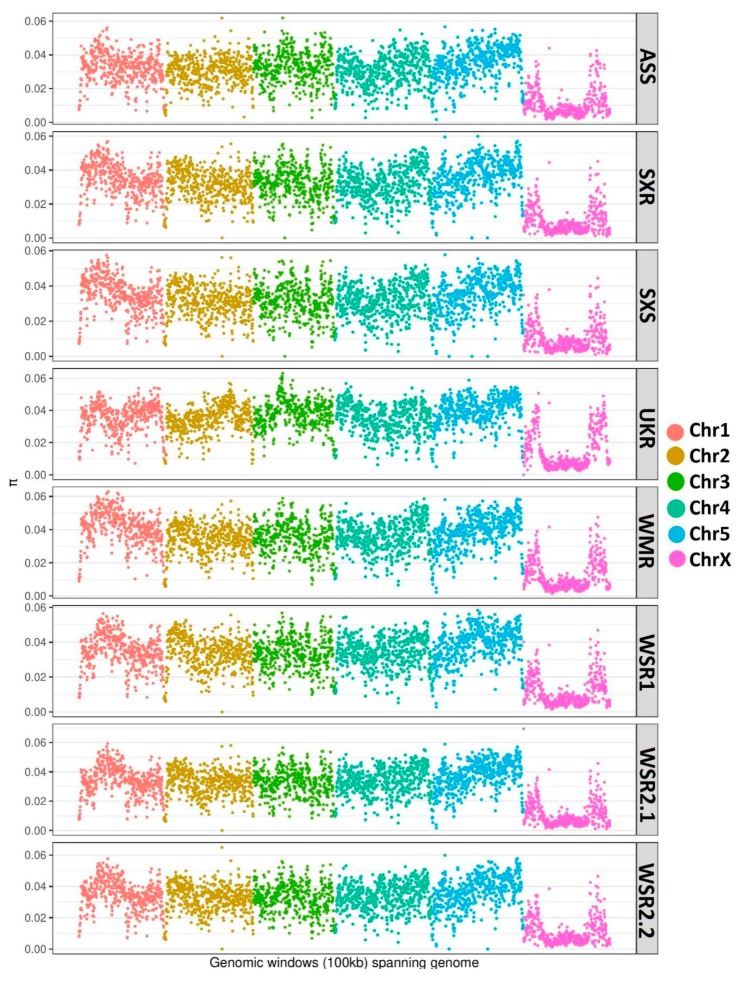
Mapping of within-strain genetic diversity across the genome in all the populations analyzed in the present study. The nucleotide diversity (pi) was calculated in 100 kb windows across the genome and mapped genome-wide in all the populations.

In order to reveal the patterns of genetic variations across the *H. contortus* populations, we characterized the genome-wide genetic diversity (both, within-strain and among-strain). Within-strain genetic diversity was assessed with nucleotide diversity estimators, Pi (π) and Watterson Theta (θ), in 100 kb windows along the genome. Both the estimators showed similar patterns of genetic diversity across the strains. High levels of nucleotide diversity (0.030 ± 0.008–0.041 ± 0.011) were observed across all the autosomes in all the populations, with the mean diversity almost three to four times higher as compared to the X chromosomal estimates (0.010 ± 0.008–0.015 ± 0.012) of nucleotide diversity (Figure 2a). Overall, highest estimate of genome-wide average nucleotide diversity (0.033 ± 0.013) was recorded in UKR and WMR strains. Lowest estimate of genome-wide average nucleotide diversity (0.029 ± 0.013) was recorded in SXR and SXS strains. These estimates are similar to that of the previous genome-wide study [40] in *H. contortus*. Overall patterns of within-strain genetic diversity were almost similar for all the strains (Figure 3).

**Figure 4:**
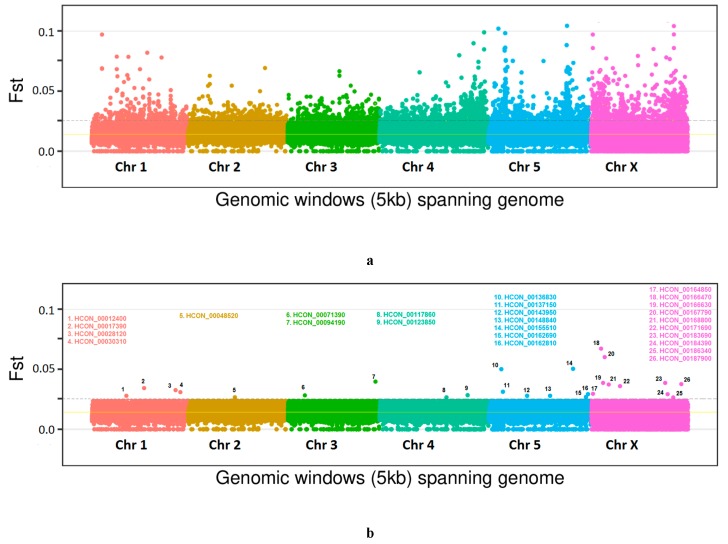
Genome-wide pairwise Fst analysis between the IVM-sensitive (SXS) and IVM-resistant (SXR) strains. Both of these strains had a very close geographic background. Elevated points are the significantly differentiated genomic windows across the genome. Yellow line shows the genome-wide average Fst cut-off. Dotted line shows the top 5% cut-off value. (**a**) All the 5 kb windows spanning the genome. (**b**) Windows containing the 26 candidate genes of IVM resistance which were selected in the present study on the basis of their functional annotations in relation to IVM resistance.

Among-strain genetic diversity was assessed by calculating genetic differentiation (Fst) in 5 kb windows along the genome. Genome-wide average Fst estimates ranged from 0.012398 to 0.090456 (Figure 2b). Pairwise comparisons of UKR strain revealed relatively higher levels of genetic differentiation than those of other strains. These values were about seven to nine times higher than those of the comparisons among the Chinese isolates, and were two times higher than those of the comparisons of ASS to Chinese isolates. The pairwise comparisons of ASS strain also showed about two times higher values then those of the comparisons among the Chinese isolates. These differences are perhaps not surprising given the geographical background and reproductive isolation of the strains. Among the Chinese isolates, the lowest genetic differentiation was observed between the SXS and SXR. These two strains had a very close geographic background, and phenotypically, one was sensitive to IVM while the other was IVM resistant. So, we next focused on this pair, and mapped the genome-wide *Fst* scores calculated in 5 kb windows spanning the genome (Figure 4). Based on pairwise *Fst* data, candidate genes were identified in the elevated outlier windows. In total, 1426 genes were laying there in top 5% cutoff (Table S3). Among these, 655 genes had either no GO terms associated with or had no *C. elegans* orthologues. So, we were unable to say anything about their IVM candidature. The remaining 771 genes were analyzed (based on associated GO terms or based on their *C. elegans* orthologues) using WormBase ParaSite [52] and *H. contortus* resources [36,51]. Based on the functional annotations of outliers by interrogating at WormBase database [38], a total of 26 genes across the genome were finally selected as candidates of IVM resistance. Details of these genes are presented in Table S4. Chromosome-wise distribution of the candidate genes included: 4 on chromosome 1, 1 on chromosome 2, 2 on chromosome 3, 2 on chromosome 4, 7 on chromosome 5, and 10 on chromosome X. The candidate genes were related to chloride channels including glutamate-gated chloride channel, oxidation-reduction process (cyt-p450), GPCR, ABC transporter and pgp, NHR (nuclear hormone receptors), Lgc (ligand-gated channel), vesicle-mediated transport, and transmembrane transportation of ions.

**Figure 5:**
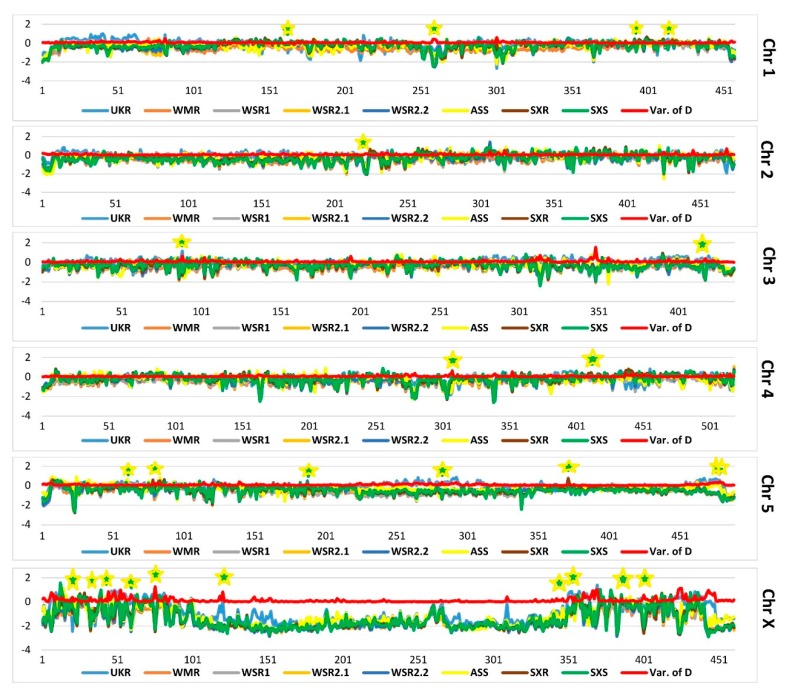
Genome-wide comparison of Tajima’s D across all the strains in the present study. Tajima’s D was calculated in 100 kb windows spanning the genome, and data was mapped chromosome wise. To emphasize the fluctuation in the allelic frequencies across the chromosomes, the variance in the mean value of Tajima’s D was calculated across the pooled data of all the populations, and mapped along with the respective chromosomal data (red line). A high variance is expected in the regions that are under the selection.

Neutrality estimator Tajima’s D was calculated in 100 kb windows along the genome using NPStat, and data was mapped chromosome wise (Figure 5). To emphasize the fluctuation in the allelic frequencies across the chromosomes, the variance in the mean value of Tajima’s D was calculated across the pooled data of all the populations, and was mapped along with the respective chromosomal data. In areas under selection, the Tajima’s D values will fluctuate among the populations, and as a result of this fluctuation, the variance of D at these areas will be higher. Thus, a high variance of D is associated with the regions that are under the selection. All candidate genes were found in peaks regions on the respective chromosomes. This further confirms that these candidate genes are under the selection.

### 3.3. RNAi Assays for Functional Validations

Two of the candidate genes, a Cyt-P450 gene (HCON_00143950) and a GPCR gene (HCON_00155510) were selected for functional validation. The selection was based on their functional annotations (HCON_00143950 is known to involve in xenobiotic metabolic process and HCON_00155510 is involved in defense response and positive regulation of gene expression) and feasibility of approach. We first got the culture of *H. contortus* larvae. The eggs hatched into L1 larval stage in about 16 h. 

Before performing the siRNA assays for candidate genes, we first checked the ingestion of siRNA by L2 larvae. A nonspecific siRNA targeting GFP was used, and strong fluorescent signals were observed in the esophagus and the intestinal lumen of the larvae (Figure S1), which show that siRNA was actively up taken by the larvae. The larvae remained viable even after 96 h of siRNA treatment, which confirmed that siRNA was nonspecific and was nontoxic for the larvae of *H. contortus*.

**Figure 6:**
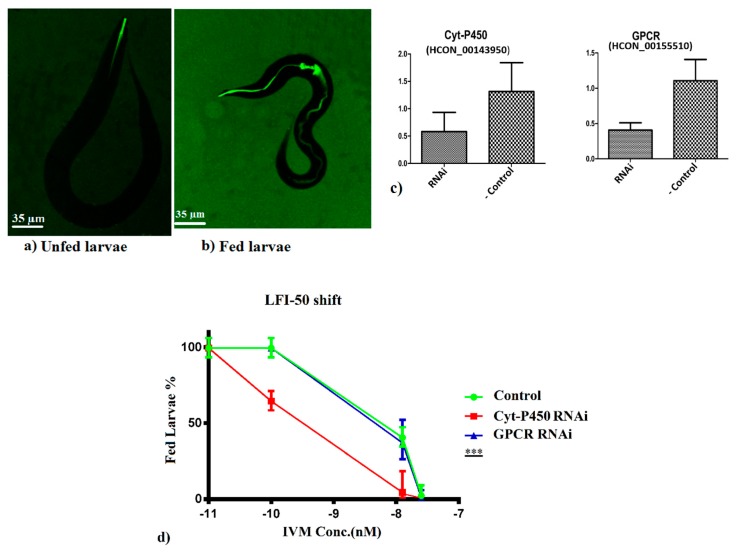
RNAi assays for functional validations. (**a**,**b**) Larval feeding of FITC-labelled *E. coli*. (**c**) Results of qPCR. Transcription level was dropped in both the genes when blocked by RNAi in comparison to RNAi negative control. (**d**) Impact of RNAi-based silencing of candidate genes on IVM efficacy against *H. contortus* L2 larvae. In the control assay, no IVM was used. Silencing of GPCR gene (HCON_00155510) showed similar response as that of control. Silencing of Cyt-P450 gene (HCON_00143950) showed a clear drop in LFI-50 against the increasing concentration of IVM. Experiments were replicated in triplicates. Values represent mean ± SD. *** *p* < 0.0001.

Next, we tested the two selected IVM-candidate genes. We first blocked these genes by RNAi, and applied IVM in serial dilutions. We then monitored the larval feeding of FITC-labelled *E. coli* (Figure 6a,b). IVM causes the paralysis of the pharynx and thus, starvation. So, when the gene associated with IVM resistance is once blocked, the resulted larvae will remain unfed (lower LFI-50). While in case of blocking a non-IVM resistant gene, the LFI-50 will be higher. The candidate gene Cyt-P450 (HCON_00143950) showed significantly low LFI-50 as compared to GPCR gene (HCON_00155510) and control sample (Figure 6d). For an IVM negative control, we used the DMSO (instead of IVM), which means that there was no IVM in that sample. For each gene, we also performed an IVM negative experiment. In that case, the gene was blocked but the larvae were actively feeding. This shows that it only has an effect (feeding inhibition) in the presence of IVM (for gene HCON_00143950). In Figure 6d at IVM concentration of zero (−11 nM for log_10_ transformation), the larvae showed active feeding. The qPCR results showed that level of blocked transcripts was significantly decreased as compared to that of negative control (Figure 6c). Overall, these data show that blocking the gene HCON_00143950 increases the sensitivity to IVM in *H. contortus*.

## 4. Discussion

The parasitic nematode *H. contortus* has a successful track record in IVM resistance. However, the genetic basis of IVM resistance is poorly resolved, and growing evidences suggest that IVM resistance in this species is likely of multigenic nature [1,22,23]. However, conventional methods or strategies could hardly characterize a few of these genes [1,21]. Use of full magnification of the genome to assess IVM resistance would provide better results. We, therefore, in this study analyzed the nature of IVM resistance in *H. contortus* at full magnification of the genome by re-sequencing the whole genome of pools of resistant and sensitive worms belonging to eight different populations. By exploiting the sampling of closely related strains together with an improved chromosome-scale assembly of the reference genome of *H. contortus*, we performed in-depth analyses, and demonstrated that knockdown of HCON_00143950 results in a decrease in larvae feeding, which may be consistent with increased sensitivity to ivermectin.

The in-depth characterization of genetic diversity showed high level of within-population genetic diversity. This is consistent with recent estimates of genome-wide studies [40,43], and also with estimates that of the earlier studies [60,61,62,63,64]. Genetic diversity in parasites helps them to avoid eradication by the host immune system [65]. *H. contortus* has shown extremely high levels of within-population genetic diversity across the globe, which is associated to its large effective population size, and provides it a high adaptive capacity [43,66,67]. The autosomal estimates of nucleotide diversity were three to four times higher than those of X-chromosomal estimates. Low level of X-chromosomal estimates of nucleotide diversity in relation to autosomes were also reported in previous genome-wide studies [40,41]. 

The complexity and genetic basis of IVM resistance has remained under much debate and discussion in connection to extent of background geographical variation and the number of genes involved in [21,33,34,35]. In order to understand the background geographical variation, we characterized the among-strain genetic diversity by pairwise Fst comparisons across the genome. The highest levels of genetic differentiation were observed in comparisons of UKR strain to others. These estimates were seven to nine times higher than those of the comparisons among the Chinese isolates, and were two times higher than those of the comparisons of ASS to Chinese isolates. Among the Chinese isolates, the lowest genetic differentiation was observed between the SXS and SXR. These two strains had a very close geographic background, and phenotypically, one was sensitive to IVM while the other was IVM resistant. So, this was the ideal pair to be subjected for further genome-wide population genomics analyses. Our results showed multiple windows on each chromosome, which were highly differentiated between the IVM-sensitive and IVM-resistant strains. Further scanning of these outliers’ windows revealed a number of genes lying there. Based on the functional annotations, GO term enrichment analysis, and *C. elegans* resources, a total of 26 genes were finally selected as candidates associated to IVM resistance. A recent genome-wide study [40] has proposed a single region of introgression that could be likely to be the major effector of IVM resistance in two populations having genetically introgressed IVM-resistant loci by genetic crosses between their IVM-sensitive and IVM-resistant parents [23]. Although, it was a single locus, its size is about 5 Mb, and it can contain as much as 360 genes. 

One of our candidate genes encodes glutamate-gated chloride channel (HCON_00148840, glc-3), and it has been analyzed as IVM-resistance-associated candidate previously [68,69,70]. The glutamate-gated chloride channel is the direct target site of IVM, where IVM binds irreversibly [71,72]. This binding causes irreversible opening of these channels, which is followed by an influx of chloride ions. The end result is a flaccid paralysis or starvation, resulting in the death of the worm [73]. Mutations in these channels have been reported to confer IVM resistance in *C. elegans* [15] and pests [69,74,75,76]. Although, we did not find any other of the previously proposed candidate genes in our set of selected candidates, we cannot discount the previous candidates too in other populations of *H. contortus*. Given the high level of genetic diversity, *H. contortus* may provide the fertile grounds for drug selection. Exposure to low levels of IVM may lead to the rapid selection and acquired tolerance against it in *H. contortus* [77]. None of the previously proposed candidate genes for IVM resistance was found in the two backcross experiments [32,40]. This scenario can be explained by highly diverse and differentiated populations of *H. contortus* having independent evolutionary histories. In such a case, the naively distributed polymorphisms in a particular population might be associated with resistance, while in another population, there could be a different polymorphism that can be associated with resistance. So in each case, there would be a different locus driving the resistance.

Majority of our candidate genes was falling under xenobiotic metabolic processes including oxidation–reduction (cytochromes P450, CYPs), ABC transporter, pgp, and NHR. Two types of anthelmintics resistance-associated mechanisms (pharmacokinetic-mediated, PKM and pharmacodynamics-mediated, PDM mechanisms) were distinguished in helminths [78]. PKM includes decreased drug uptake, an accelerated drug efflux, and an increased drug inactivation, whereas PDM encompasses structural and numerical changes in the drug target macromolecules that result in the reduction of drug efficacy. If a drug fails to bind to altered drug targets, a higher dose of drug will not be effective too [79]. PKM detoxification pathways are accomplished in three successive phases of xenobiotic metabolism. Phase I includes the modifying processes, such as oxidation, reductions, or hydrolysis of drugs. Phase II consists on conjugation reactions where xenobiotics or their phase I metabolites are conjugated with other compounds, such as amino acids, glucuronic acid, and sulphates. Phase III includes the transporter proteins that carry out the active transport of xenobiotics or their metabolites and conjugates through the membrane. CYPs involve in phase I of xenobiotic metabolism where they perform the biotransformation of a vast number of xenobiotics [78,80,81]. CYPs are accounted for the biotransformation of about 90% of the drugs [82]. While CYPs are widely distributed in nature, their presence in helminths is ignored for a long time [83,84]. However, this view has been changed with the discovery of CYPs-encoding genes in *C. elegans*, where more than 80 CYP proteins are present [85,86], and these have been shown to be xenobiotics inducible [85,87,88,89,90]. The CYPs family have also been characterized recently in *H. contortus* [91,92]. ABC transporter and pgp fall under the phase III of xenobiotic detoxification process. They transport the hydrophilic metabolites across the membrane at the expense of ATP [78,93]. The role of ABC transporter in drug resistance has been reviewed [94]. About 60 genes encode the ABC transporter in *C. elegans* [86]. The first pgp in helminths was also described in *C. elegans* [95], and then, they were discovered in other flukes and nematodes [96]. In *H. contortus*, pgps are predominantly expressed along the intestinal tract and are considered to have a role in IVM resistance [97,98] and moxidectin resistance [99]. It has also been demonstrated in mice that IVM induced the expression of CYPs and ABC transporters [100]. NHRs when bind to a xenobiotic act as transcription factors and induce the expression of genes involved in xenobiotic detoxification [101]. They regulate the CYPs families in mammals [102,103]. They have been found to be involved in xenobiotic metabolism and detoxification in *C. elegans* [104], fruit fly [105], and *H. contortus* [1]. Some of our candidate genes fall under transmembrane transportation of ions and GPCRs. The transmembrane ion channels control the potential across the membrane and thus involve in signaling [106]. GPCRs are highly druggable and are about 50% of human drugs are directed at these proteins [107,108,109]. They have been reported having broad anthelmintic activity in *H. contortus* [110] and other helminths [109]. Recently, a G-protein-gated channel was found being directly activated by IVM [111]. Many neurotransmitters also act through GPCRs [106]. The biogenic amines including 5-HT, octopamine, tyramine, and dopamine act at GPCRs [112], and chloride channels in nematodes are gated by dopamine, tyramine, and 5-HT. In *C. elegans*, 5-HT and dopamine have roles in feeding and locomotion [113]. The classical neurotransmitters, GABA and acetylcholine, also act as GPCRs and regulate the locomotion in worms [114].

Finally, we selected two (HCON_00143950, Cyt-P450 and HCON_00155510, GPCR) of the candidate genes to be subjected for functional validation using RNAi-based assays in *H. contortus*. This selection was based on the functional annotations of candidates and the feasibility of the validation assay (both for the candidate genes and the available resources at our lab). The Cyt-P450 is known to involve in xenobiotic metabolic processes, while the GPCRs involve in defense responses and positive regulation of gene expression. Although, xenobiotic metabolism has been widely studied in mammals, the extent of xenobiotic metabolic processes involved in nematodes remain to be resolved [115,116,117]. We specifically aimed to evaluate the potential involvement of these candidate genes in IVM tolerance in *H. contortus*. The CYP gene (HCON_00143950) significantly reduced the feeding in larvae when it was silenced by RNAi. The CYPs show increased expression when exposed to xenobiotic [115,116]. In *C. elegans*, the overexpression of cyps35 has been reported against albendazole [115] and TBZ [116] exposure, while TBZ was metabolized by CYP35D1 [118]. The gene HCON_00143950 is an orthologue of *C. elegans* CYP33. The CYP33 has been shown to be xenobiotic inducible in *C. elegans* [85]. CYPs in *H. contortus* have also been studied in relation to their inhibitors and inducers [119,120,121,122].

A comparative genomics approach using full magnification of genome followed by functional validation assay enabled us here to successfully screen and identify IVM-resistance-associated genes in *H. contortus*. Although we successfully validated the candidate gene using RNAi, a genetic manipulation-based assay using *C. elegans* is expected to provide further insights. 

## 5. Conclusions

In summary, we applied a comparative genomics approach at the full magnification of genome to assess the genome-wide diversity in pairwise comparisons among the drug-resistant and -sensitive worms, and revealed the IVM-resistance-associated candidate genes in a blood-feeding parasitic nematode *H. contortus*. These genes were predicted based on analyses performed on the genomes of genetically very close strains, and in that we have tried to minimize the amount of background variation that often confounds analyses of different and geographically diverse strains. Our analyses also revealed a previously known IVM resistance-associated candidate gene HCON_00148840, glc-3. Finally, the CYP gene (HCON_00143950) significantly reduced the feeding in larvae when it was silenced by RNAi, which shows that this gene (when blocked) may increase the sensitivity to IVM in *H. contortus*. These results further enhance our understanding on the IVM resistance and continue to provide more evidence in favor of multigenic nature of IVM resistance. Future screening and characterization of the candidates might provide deeper insights and better resolution of the IVM resistance. Consequently, the implication will further refine the management strategies of the nematodes of veterinary and medical importance.

## Figures and Tables

**Table 1:** Details of samples.

Pool	Strain Name	Code	IVM-Phenotype	Pool Size
1	Wu Meng-R	WMR	Resistant	50
2	Wu Shen-R1	WSR1	Resistant	50
3	Shan Xi-R	SXR	Resistant	50
4	Shan Xi-S	SXS	Sensitive	50
5	UK-R	UKR	Resistant	50
6	Wu Shen-R2.1	WSR2.1	Resistant	50
7	Wu Shen-R2.2	WSR2.2	Resistant	50
8	Australian-S	ASS	Sensitive	50

## Supplementary Materials

The following are available online at https://www.mdpi.com/2073-4425/11/4/367/s1; Figure S1: ingestion of siRNA by L2 larvae of *Haemonchus contortus* in the present study; Table S1: details of siRNA sequences used in the present study; Table S2: primers for qPCR assays used in the present study; Table S3: details of outlier genes in the present study; Table S4: details of candidate genes putatively associated to IVM resistance in *H. contortus*. Additional File F1: Whole genome re-sequencing.
