# Supplementary material for: A Whole Genome Re-Sequencing Based GWA Analysis Reveals Candidate Genes Associated with Ivermectin Resistance in Haemonchus contortus
Source: Genes (Basel). 2020 Mar 28;11(4):367. doi: 10.3390/genes11040367 (PMC7230667; doi:10.3390/genes11040367)
Supplement: Supplementary file 1 [file genes-11-00367-s001.zip › genes-728242-supplementary/Supplementary Figures and Tables.docx]

**Supplementary Figures and Tables**

| 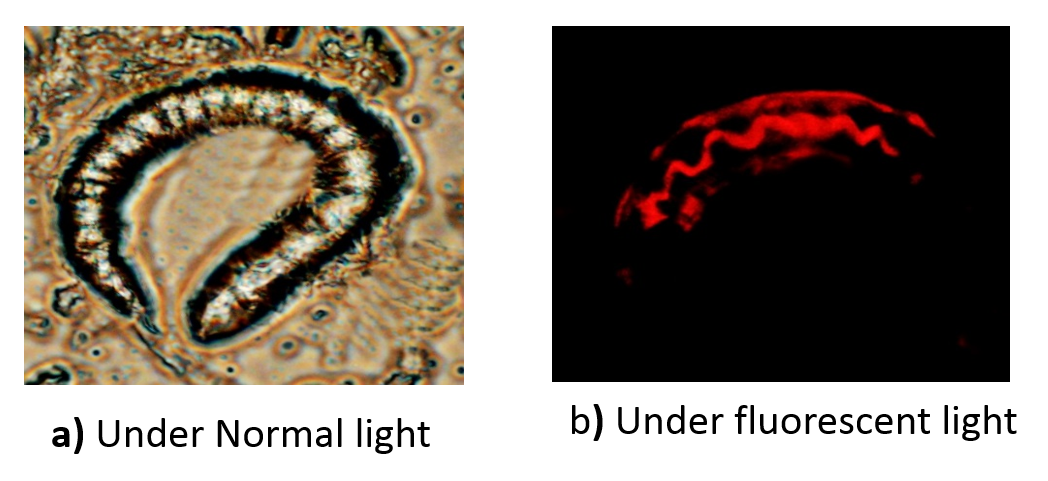 |
| --- |

**Figure S1.** Ingestion of siRNA by L2 larvae of Haemonchus contortus in present study.

**Table S1.** Details of siRNA sequences used in present study.

| **Gene ID** | **Sens (5'-3')** | **Antisense (5'-3‘)** |
| --- | --- | --- |
| HCON_00143950 | AUUGACAUGCAGGACAUCAdTdT | UGAUGUCCUGCAUGUCAAUdTdT |
| HCON_00155510 | UACUCAGGACCGCAAACGUdTdT | ACGUUUGCGGUCCUGAGUAdTdT |
| Non-specific (gfp) | CUACCUGUUCCAUGGCCAAdTdT | UUGGCCAUGGAACAGGUAGdTdT |

**Table S2.** Primers for qPCR assays used in present study

| **Gene ID** | **Forward (5'-3')** | **Reverse (5'-3‘)** |
| --- | --- | --- |
| HCON_00143950 | TGGCTTTGATTCTTTTTATGCTTCCC | AACGTAGTCATTTGTATGCTCTGAGT |
| HCON_00155510 | ATCGATAAGCAAATCAAGCAGGGAG | GTTGTGTCATTTCATCTTCGGTAAACCC |
| GAPDH HCON_00063590 (internal control) | GTGTGAACCACGAGACCTACA | TATCGTCCATGCTAGCTGGTT |

**Table S4.**  Details of candidate genes putatively associated to IVM resistance in H. contortus.

| **S/No.** | **Chromosome** | **Gene ID** | **Description** |
| --- | --- | --- | --- |
| 1 | Chr-5 | HCON_00136830 | Transmembrane transport; cation; chloride symporter activity; nkcc-1; Na-K-Cl symporter activity |
| 2 | Chr-5 | HCON_00137150 | mod-1; Serotonin-gated chloride channel subunit ; extracellular ligand-gated ion channel activity; transmembrane signalling |
| 3 | Chr-5 | HCON_00143950 | cyt-P450; oxidation-reduction process; reduction of molecular oxygen; heme binding; cyp-33C1; Cytochrome P450 family; xenobiotic metabolic process |
| 4 | Chr-5 | HCON_00148840 | Glutamate-gated chloride channel-α sub unit; extracellular ligand-gated ion channel activity; glc-3 ; Glutamate-gated chloride channel subunit |
| 5 | Chr-5 | HCON_00155510 | GPCR; G-protein beta/gamma-subunit complex binding; gpa-2 ; involved in defence response to Gram-negative bacterium; positive regulation of gene expression |
| 6 | Chr-5 | HCON_00162690 | Ion transmembrane transport; extracellular ligand-gated ion channel activity; lgc-44; Ligand-Gated ion Channel; chloride channel activity; |
| 7 | Chr-5 | HCON_00162810 | Vesicle-mediated transport; cni-1; regulation of NMDA receptor activity-modulate glutamate receptor activity; AMPA selective glutamate receptor activity |
| 8 | Chr-1 | HCON_00012400 | Zinc ion binding; Dystrobrevin-1; acetylcholine transmembrane transporter activity; drug transmembrane transport; |
| 9 | Chr-1 | HCON_00017390 | NHR-49; Nuclear hormone receptor family member nhr-49; regulation of transcription, DNA-templated; DNA-binding transcription factor activity; |
| 10 | Chr-1 | HCON_00028120 | NHR-62; regulation of transcription, DNA-templated; DNA-binding transcription factor activity |
| 11 | Chr-1 | HCON_00030310 | Extracellular ligand-gated ion channel activity; transmembrane signalling receptor activity; lgc-51; Ligand-Gated ion Channel; chloride channel activity |
| 12 | Chr-2 | HCON_00048520 | chloride channel activity; extracellular ligand-gated ion channel activity; lgc-45 Ligand-Gated ion Channel; chloride transmembrane transport |
| 13 | Chr-3 | HCON_00071390 | Voltage-gated chloride channel activity; chloride transport; transmembrane transport chloride ions |
| 14 | Chr-3 | HCON_00094190 | ABC transporter domain containing protein; transmembrane transport; ATPase activity, coupled to transmembrane movement of substances |
| 15 | Chr-4 | HCON_00117860 | nhr-22; regulation of transcription, DNA-templated; zinc ion binding |
| 16 | Chr-4 | HCON_00123850 | Acetylcholine-gated chloride channel ACC-2; extracellular ligand-gated ion channel activity; chloride transmembrane transport |
| 17 | Chr-X | HCON_00164850 | NHR daf-12 ; regulation of transcription, DNA-templated; Nuclear hormone receptor family member daf-12; regulation of pharyngeal pumping |
| 18 | Chr-X | HCON_00166470 | NHR-35; regulation of transcription, DNA-templated; |
| 19 | Chr-X | HCON_00166630 | NHR-14; regulation of transcription, DNA-templated |
| 20 | Chr-X | HCON_00167790 | Lgc-53; ion transmembrane transport; extracellular ligand-gated ion channel activity; chloride transmembrane transport |
| 21 | Chr-X | HCON_00168800 | pgp-10; transmembrane transport; p-Glycoprotein related; ABC transporter containing domain |
| 22 | Chr-X | HCON_00171690 | NHR-111; regulation of transcription, DNA-templated; steroid hormone receptor activity |
| 23 | Chr-X | HCON_00183690 | Extracellular ligand-gated ion channel activity; lgc-47; Ligand-Gated ion Channel; chloride transmembrane transport |
| 24 | Chr-X | HCON_00184390 | NHR-3; regulation of transcription, DNA-templated; sequence-specific DNA binding |
| 25 | Chr-X | HCON_00186340 | GPCR; G protein-coupled receptor signalling pathway; GABA; response to drug; |
| 26 | Chr-X | HCON_00187900 | NHR-40; regulation of transcription, DNA-templated |
