# Supplementary material for: A Whole Genome Re-Sequencing Based GWA Analysis Reveals Candidate Genes Associated with Ivermectin Resistance in Haemonchus contortus
Source: Genes (Basel). 2020 Mar 28;11(4):367. doi: 10.3390/genes11040367 (PMC7230667; doi:10.3390/genes11040367)
Supplement: Supplementary file 1 [file genes-11-00367-s001.zip › genes-728242-supplementary/Additional file F1.docx]

**Additional file F1: Whole genome re-sequencing**

| **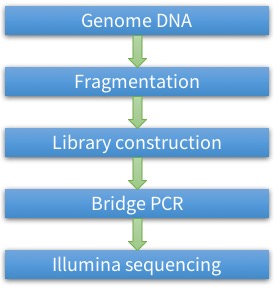** |
| --- |
| **Fig F1.1** Library construction flow chart |

| 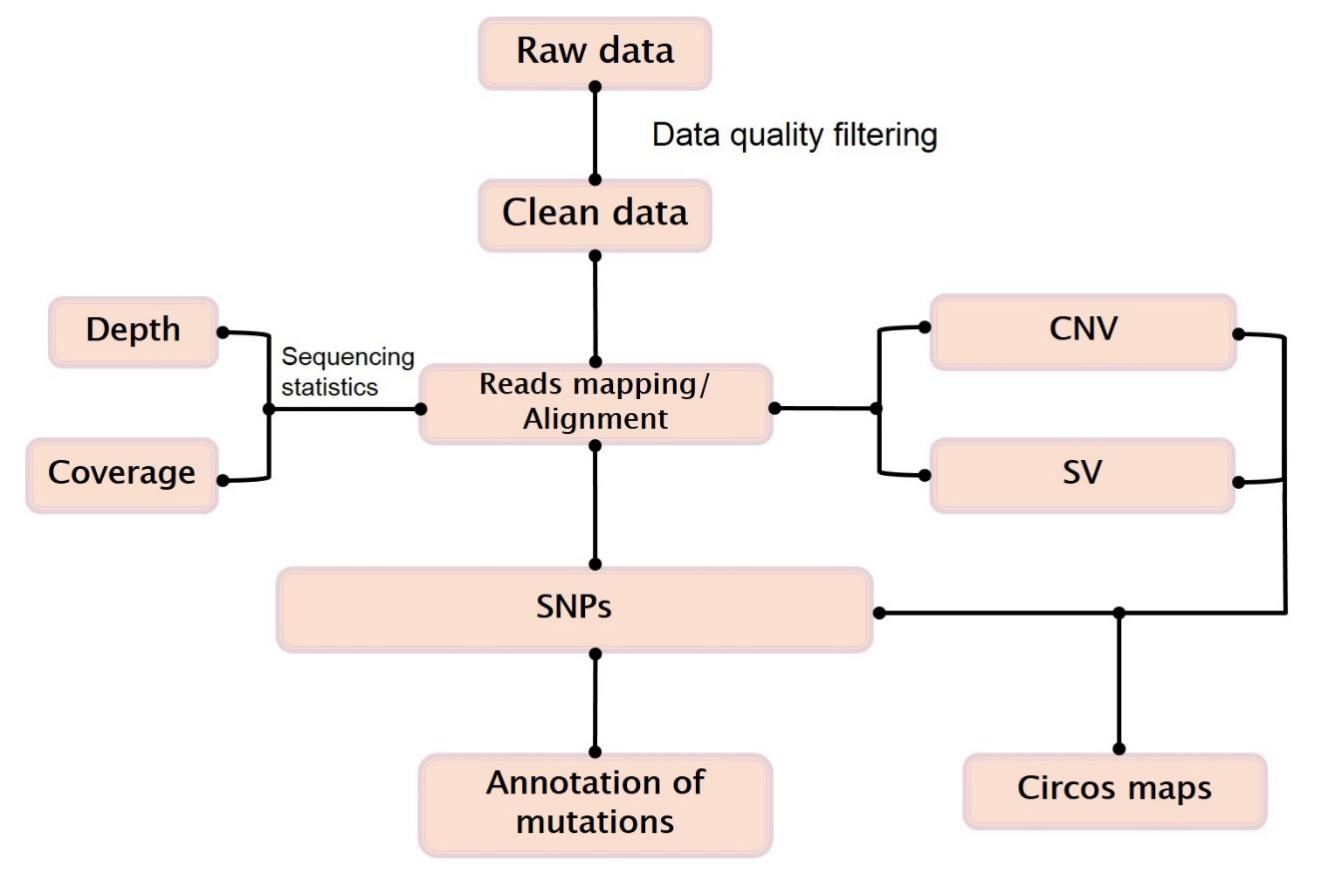 |
| --- |
| **Fig F1.2** Schematic of bioinformatics analysis of sequenced data |

**Table F1.1** Summary of quality control results

| Samples | Raw reads | Clean reads | Clean reads % | Raw base | Clean base | Clean base % | GC content | >*Q20 | >Q30 |
| --- | --- | --- | --- | --- | --- | --- | --- | --- | --- |
| Wu Meng R | 149057116 | 145438204 | 97.57% | 22358567400(22.36Gb) | 21590660816(21.59Gb) | 96.57% | 43.16% | 98.18% | 95.51% |
| Wu Shen R1 | 168165874 | 164113124 | 97.59% | 25224881100(25.22Gb) | 24422788164(24.42Gb) | 96.82% | 42.36% | 98.30% | 95.73% |
| Shan Xi R | 159226192 | 155358102 | 97.57% | 23883928800(23.88Gb) | 23105296970(23.11Gb) | 96.74% | 43.65% | 98.28% | 95.66% |
| Shan Xi S | 147422772 | 144177648 | 97.80% | 22113415800(22.11Gb) | 21431266696(21.43Gb) | 96.92% | 43.56% | 98.38% | 95.88% |
| UKR | 153195144 | 149940954 | 97.88% | 22979271600(22.98Gb) | 22273981960(22.27Gb) | 96.93% | 43.01% | 98.33% | 95.80% |
| Wu Shen R2.1 | 153740218 | 149684194 | 97.36% | 23061032700(23.06Gb) | 22244423124(22.24Gb) | 96.46% | 42.52% | 98.10% | 95.26% |
| Wu Shen R2.2 | 138982638 | 135145684 | 97.24% | 20847395700(20.85Gb) | 20087707712(20.09Gb) | 96.36% | 42.47% | 98.08% | 95.25% |
| Australian S | 141110898 | 137815496 | 97.66% | 21166634700(21.17Gb) | 20483830352(20.48Gb) | 96.77% | 43.13% | 98.15% | 95.40% |

* *Q* = Phred quality score: Q20, Base calling accuracy of 0.99; Q30, Base calling accuracy of 0.999

**Table F1.2** Statistics of genome-wide alignment

| Samples | Total reads | Duplicates | Duplicates percentage | Total reads after removing duplicates | Mapped reads | Mapped Rate | Mean coverage | Mean mapping quality | 1X coverage | 10X coverage |
| --- | --- | --- | --- | --- | --- | --- | --- | --- | --- | --- |
| Wu Meng R | 145438204 | 19545010 | 13.44% | 125893194 | 112981184 | 89.74% | 53.3053X | 48.9625 | 96.56% | 91.35% |
| Wu Shen R1 | 164113124 | 19848808 | 12.09% | 144264316 | 106003892 | 73.48% | 49.594X | 49.132 | 96.34% | 90.08% |
| Shan Xi R | 155358102 | 20940373 | 13.48% | 134417729 | 113527974 | 84.46% | 53.8084X | 49.5452 | 96.20% | 91.01% |
| Shan Xi S | 144177648 | 19795188 | 13.73% | 124382460 | 103094139 | 82.88% | 48.6327X | 49.5305 | 96.25% | 90.13% |
| UKR | 149940954 | 19083403 | 12.73% | 130857551 | 118443275 | 90.51% | 55.8903X | 49.3459 | 96.56% | 90.61% |
| Wu Shen R2.1 | 149684194 | 17228106 | 11.51% | 132456088 | 98832958 | 74.62% | 46.344X | 49.081 | 96.25% | 89.79% |
| Wu Shen R2.2 | 135145684 | 15189065 | 11.24% | 119956619 | 91672737 | 76.42% | 43.0627X | 49.0748 | 96.18% | 89.13% |
| Australian S | 137815496 | 19697420 | 14.29% | 118118076 | 107546422 | 91.05% | 51.4505X | 49.7374 | 96.78% | 91.78% |

**Table F1.3** Statistics of SNPs annotation

| Mutation type | | Wu Meng R | Wu Shen R1 | Shan Xi R | Shan Xi S | UKR | Wu Shen R2.1 | Wu Shen R2.2 | Australian S |
| --- | --- | --- | --- | --- | --- | --- | --- | --- | --- |
| CDS | | 53159 | 58886 | 61277 | 62461 | 82076 | 60948 | 60778 | 50896 |
|  | Synonymous | 36344 | 40681 | 41918 | 42922 | 58983 | 41968 | 41795 | 35290 |
|  | Missense | 16332 | 17697 | 18795 | 18992 | 22523 | 18407 | 18442 | 15152 |
|  | Stop gained | 280 | 283 | 339 | 327 | 336 | 334 | 301 | 255 |
|  | Stop lost | 120 | 133 | 140 | 127 | 133 | 138 | 134 | 121 |
|  | Others | 83 | 92 | 85 | 93 | 101 | 101 | 106 | 78 |
| UTR5' | | 3834 | 4163 | 4269 | 4553 | 5796 | 4605 | 4497 | 3538 |
| UTR3' | | 948 | 990 | 993 | 1035 | 1411 | 956 | 1015 | 766 |
| Intronic | | 292679 | 326473 | 338354 | 344293 | 412794 | 336972 | 339232 | 275746 |
| Splicing | | 9420 | 10537 | 10683 | 11058 | 14558 | 10895 | 10805 | 8944 |
| Upstream | | 321522 | 359484 | 365795 | 376085 | 434298 | 369302 | 368489 | 303225 |
| Downstream | | 202402 | 226249 | 233492 | 238784 | 279498 | 232330 | 230497 | 190953 |
| Intergenic | | 251153 | 282448 | 291420 | 299821 | 343312 | 287826 | 293336 | 245541 |
| **Total** | | **1135117** | **1269230** | **1306283** | **1338090** | **1573743** | **1303834** | **1308649** | **1079609** |

**Table F1.4** Statistics of nucleotide substitutions

| Nt. Substitution | Wu Meng R | Wu Shen R1 | Shan Xi R | Shan Xi S | UKR | Wu Shen R2.1 | Wu Shen R2.2 | Australian S |
| --- | --- | --- | --- | --- | --- | --- | --- | --- |
| A>C | 54593 | 60352 | 61791 | 63272 | 74917 | 61611 | 61809 | 51408 |
| A>G | 175079 | 196020 | 201970 | 207346 | 242846 | 201388 | 202609 | 166409 |
| A>T | 71041 | 78940 | 81129 | 83407 | 98493 | 81170 | 81431 | 67299 |
| C>A | 46672 | 52694 | 54147 | 55484 | 64512 | 54194 | 54201 | 44638 |
| C>G | 41588 | 46329 | 47805 | 48888 | 56674 | 47488 | 47652 | 39628 |
| C>T | 180960 | 202439 | 208405 | 213787 | 251084 | 208550 | 209690 | 172232 |
| G>A | 179328 | 200859 | 206960 | 212229 | 249410 | 207108 | 207315 | 170752 |
| G>C | 40583 | 45273 | 46476 | 47608 | 56040 | 46164 | 46370 | 38607 |
| G>T | 47514 | 53228 | 55077 | 56239 | 65637 | 54858 | 54961 | 45629 |
| T>A | 69941 | 77628 | 79749 | 81451 | 97290 | 79659 | 80202 | 66427 |
| T>C | 173678 | 195664 | 201391 | 206138 | 242114 | 200376 | 201280 | 165608 |
| T>G | 54140 | 59804 | 61383 | 62241 | 74726 | 61268 | 61129 | 50972 |
| **Total** | **1135117** | **1269230** | **1306283** | **1338090** | **1573743** | **1303834** | **1308649** | **1079609** |
| Transitions | 709045 | 794982 | 818726 | 839500 | 985454 | 817422 | 820894 | 675001 |
| Transversions | 426072 | 474248 | 487557 | 498590 | 588289 | 486412 | 487755 | 404608 |

**Table F1.5** Statistics of InDel annotation

| Mutation type | | Wu Meng R | Wu Shen R1 | Shan Xi R | Shan Xi S | UKR | Wu Shen R2.1 | Wu Shen R2.2 | Australian S |
| --- | --- | --- | --- | --- | --- | --- | --- | --- | --- |
| CDS | | 2154 | 2351 | 2505 | 2498 | 2475 | 2411 | 2414 | 2110 |
|  | Inframeshift | 298 | 322 | 351 | 353 | 420 | 342 | 345 | 282 |
|  | Frameshift | 1848 | 2015 | 2133 | 2130 | 2042 | 2050 | 2053 | 1819 |
|  | Stop gained | 5 | 8 | 16 | 10 | 10 | 13 | 11 | 6 |
|  | Stop lost | 1 | 3 | 3 | 2 | 0 | 4 | 2 | 1 |
|  | Others | 2 | 3 | 2 | 3 | 3 | 2 | 3 | 2 |
| UTR5' | | 711 | 813 | 862 | 826 | 1042 | 879 | 830 | 719 |
| UTR3' | | 155 | 147 | 151 | 159 | 202 | 151 | 158 | 115 |
| Intronic | | 53757 | 61344 | 62677 | 64022 | 69049 | 63228 | 63156 | 50510 |
| Splicing | | 1477 | 1695 | 1738 | 1775 | 1960 | 1804 | 1750 | 1410 |
| Upstream | | 58267 | 67062 | 67274 | 69565 | 70832 | 67982 | 68004 | 53939 |
| Downstream | | 38240 | 43883 | 43992 | 45367 | 46936 | 44191 | 44234 | 35121 |
| Intergenic | | 45358 | 52089 | 53074 | 54732 | 54270 | 53231 | 53926 | 43088 |
| **Total** | | **200119** | **229384** | **232273** | **238944** | **246766** | **233877** | **234472** | **187012** |
| Deletions | | 103061 | 117906 | 119569 | 123055 | 126573 | 120497 | 120685 | 96444 |
| Insertions | | 97058 | 111478 | 112704 | 115889 | 120193 | 113380 | 113787 | 90568 |

| 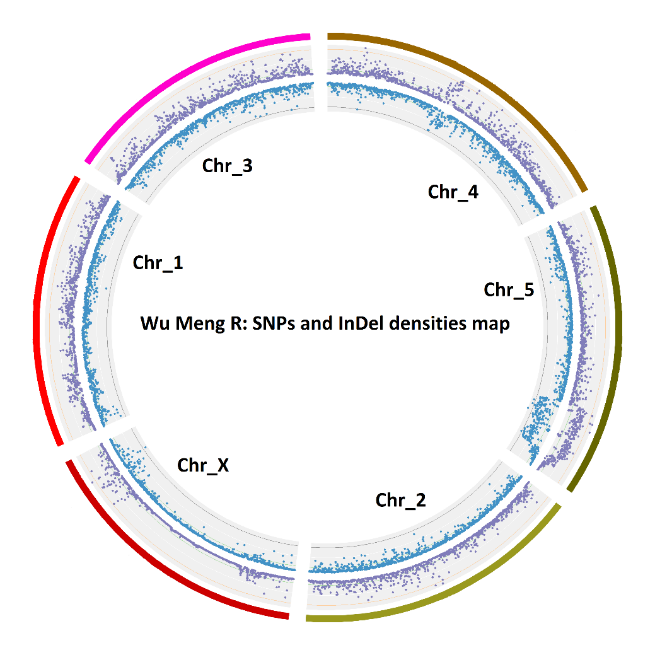 | 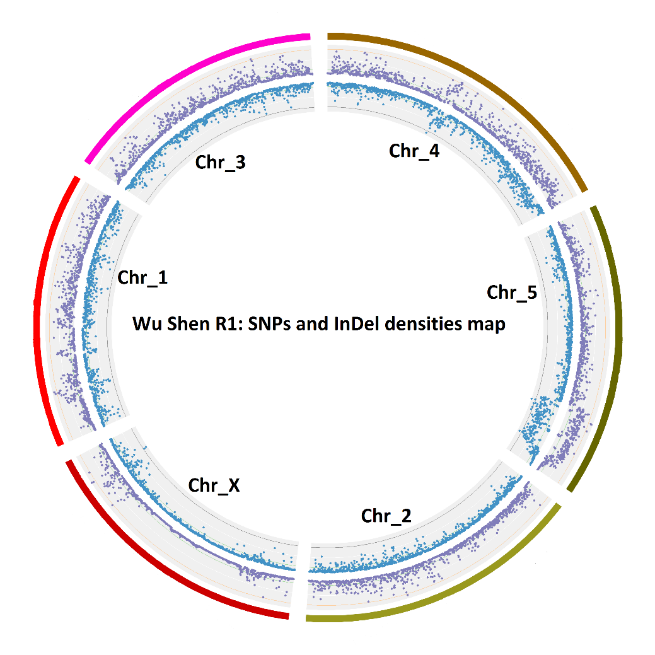 |
| --- | --- |
| 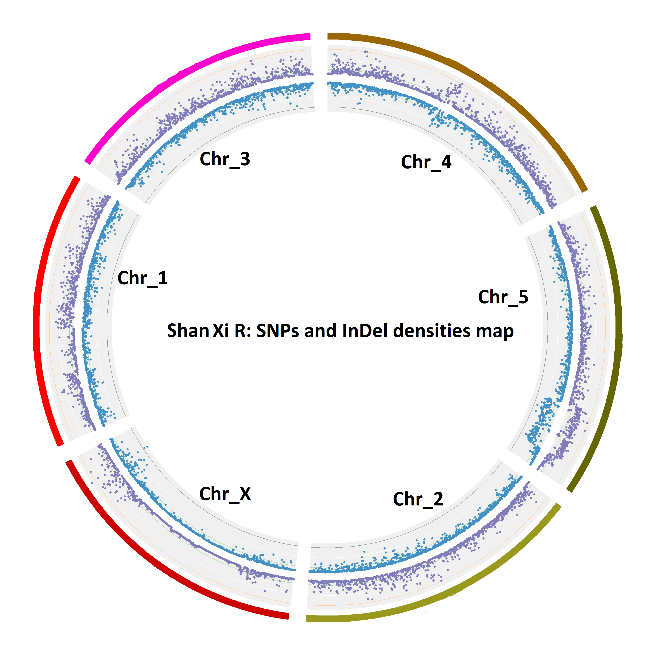 | 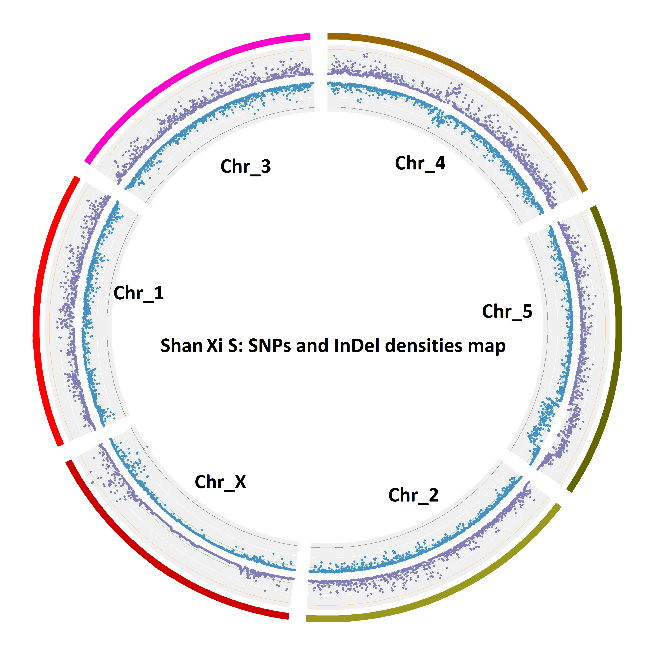 |
| 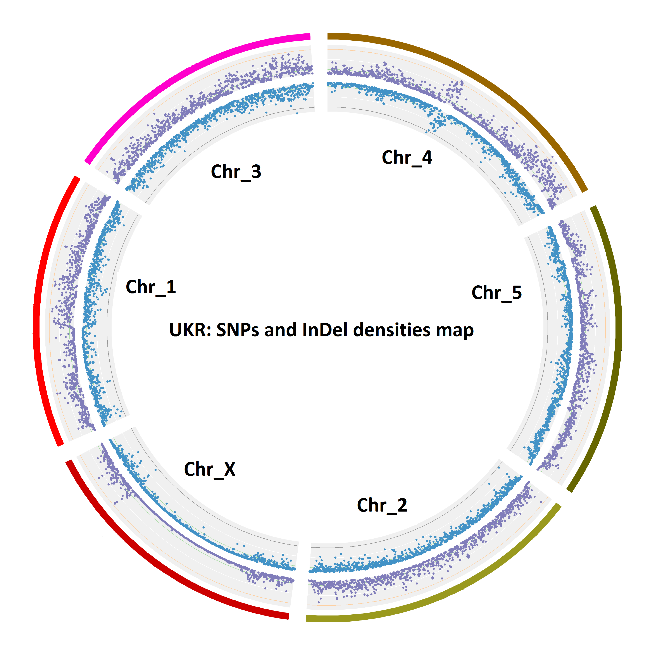 | 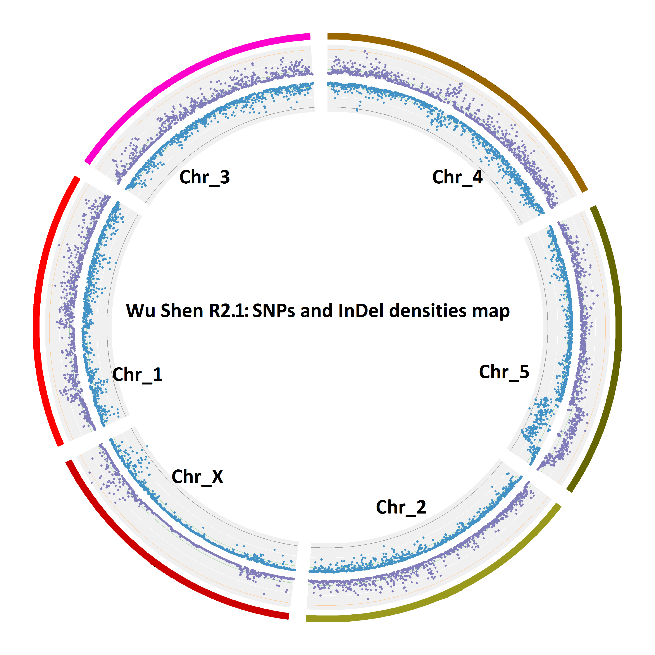 |
| 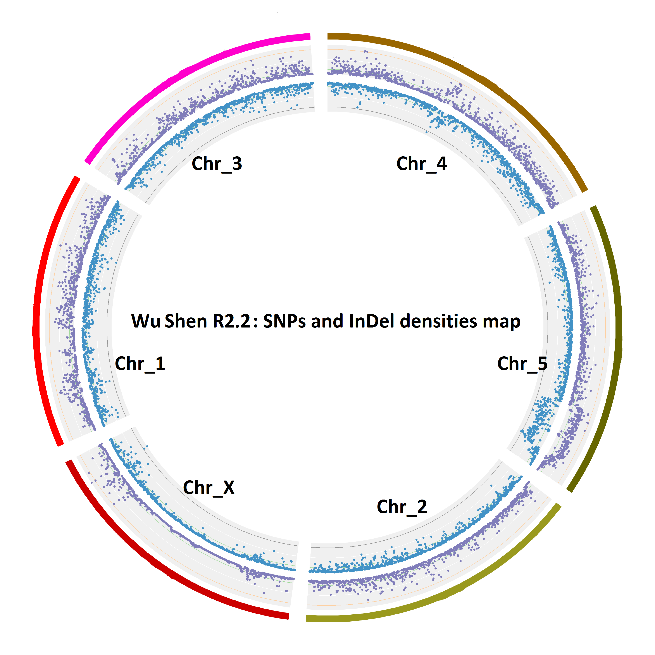 | 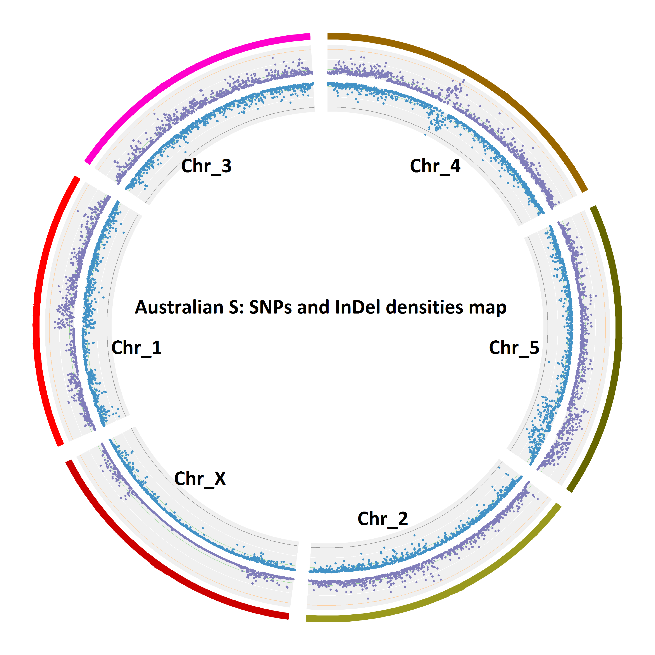 |

**Fig F1.3** Genomic variation Circos diagrams. 1st circle: chromosome; Circle 2: Dark purple dots indicate SNP density, and larger values are towards outer ring; Circle 3: Dark blue dots indicate the density of the InDel, and larger values are towards inner ring. Chromosome size increases in clockwise.
